# Getting ready for the winter: Timing and determinants of molt in an alpine ungulate

**DOI:** 10.1002/ece3.4970

**Published:** 2019-02-14

**Authors:** Florent Déry, Sandra Hamel, Steeve D. Côté

**Affiliations:** ^1^ Département de biologie and Centre d’études nordiques Université Laval Québec Québec Canada; ^2^ Department of Arctic and Marine Biology, Faculty of Biosciences, Fisheries, and Economics UiT The Arctic University of Norway Tromsø Norway

**Keywords:** aging, allocation, hair growth, mountain goat, resources availability

## Abstract

Because growth of new hairs entails energetic costs, individual condition and access to food should determine the timing of molt. Previous studies on the timing of molt in ungulates have mostly focused on the influence of age class and reproductive status, but the effects of body condition and environmental phenology have not been evaluated. Our goal was to assess how intrinsic traits and environmental conditions determine the timing of winter coat shedding in a mountain goat population monitored for 27 years. The date of molt completion followed a U shape with age, suggesting that senescence occurs in terms of the molting process in mountain goats. Juveniles of both sexes delayed molting in a similar fashion, but molt timing differed between sexes during adulthood. Males molted progressively earlier until reaching age when reproduction peaked, after which they started delaying molting again. Females reached earliest molt dates at age of first reproduction and then progressively delayed molt date. Lactating females molted 10 days later than barren females on average, but this only occurred in females in good condition. Thus, although it has been shown that reproduction delays molt in ungulates, our results indicate that body condition can override this effect. Overall, our results revealed that access to both extrinsic and intrinsic resources is one of the key mechanisms driving molting processes in a mammalian herbivore.

## 1 INTRODUCTION

Pelage provides concealment, thermoregulation, and protection, and is therefore a key component for mammals facing harsh climatic conditions in northern environments (Caro, 2005; Cowan & Raddi, 1972; Zimova, Mills, Lukacs, & Mitchell, 2014; Zimova, Mills, & Nowak, 2016). Several species exposed to large temperature variations in their environment grow and molt one or two annual coats (one for summer and one for winter; Maurel, Coutant, Boissin‐Agasse, & Boissin, 1986; Beltran, Burns, & Breed, 2018). Growing and molting new hairs involve energetic costs (Boyd, Arnbom, & Fedak, 2012; Neuhaus, 2000), particularly after winter, a period associated with high energetic expenses and limited resources for herbivores (Stewart, Bowyer, Dick, Johnson, & Kie, 2005). Molt timing has often been studied in birds (Bridge, 2006; Rohwer, Ricklefs, Rohwer, & Copple, 2009), but fewer studies have been done in mammalian species. As a result, we know much less about the factors driving molt timing in mammals (Beltran et al., 2018). Most studies on mammals assessed histological processes and provided descriptive analyses of hair follicle growth (Blix et al., 2015; Cuyler & Øritsland2002a, 2002b; Nixon, Gurnseyb, Betteridgec, Mitchellc, & Welchc, 1991). Some studies, however, also investigated the factors affecting molt timing, such as age class, reproductive status (Cowan & Raddi, 1972; Fraser, Longstaffe, & Fenton, 2013; Zimova et al., 2018), individual condition, or access to food resources (Heydon, Milne, Brinklow, & Loudon, 1995; Macdonald & Stewart, 1997). In addition, photoperiod has been found to influence mammalian molt processes (Lynch, 1973; Nixon, Ashby, Saywell, & Pearson, 1995), especially in high‐latitude environments (Ling, 1970). Photoperiod can also interact with temperature to affect molt processes (Ling, 1970; Mills et al., 2013; but see Hammond, 1952), with colder temperatures delaying the onset of molt (Rust, 1962). Although these studies have been insightful, we still lack a comprehensive knowledge on the overall mechanisms shaping the molting process in temperate and arctic/alpine mammals.

Extrinsic parameters such as photoperiod and temperature are known to affect the growth of winter coat as they increase the production of hormones (i.e., melatonin increases as day length is shortening, which inhibits prolactin production; Zimova et al., 2018) that stimulate the development of hair follicles (Johnson, 1981; Lynch, 1973; Nixon et al., 1995). New hair growth should also be related to environmental phenology in highly seasonal systems because winter limits nutrient intake (Stewart et al., 2005). As an example, hair growth often overlaps periods of high resource availability and quality (Cowan & Raddi, 1972; Nixon et al., 1995) in temperate and arctic/alpine mammals. Despite this, little attention has been given to direct effect of vegetation phenology on molt in mammals. In birds however, an increase in food abundance accelerated timing of pre‐alternate molt in swamp sparrows (*Melospiza georgiana*; Danner, Greenberg, Danner, & Walters, 2015). In four nectar‐feeding bird species, timing of molt was also positively correlated with flower abundance (Wolfe, Ralph, & Wiegardt, 2017). Therefore, in habitats with short periods of resources availability, we expect harsh climatic conditions that delay spring green‐up to delay molt because hair growth is likely related to the timing of the peak in vegetation quality.

In addition to direct environmental effects, individual characteristics could affect the timing of molt. Because growth of new hair implies energetic costs, molt completion can be delayed in individuals in poor condition (Beltran et al., 2018; Macdonald & Stewart, 1997; Zimova et al., 2018). Individual traits linked to body condition such as age, reproductive status, social rank, or body mass are therefore likely to influence the timing and duration of the molt. For instance, juveniles face higher energy expenditures associated with body growth and maintenance than adults (Gaillard et al., 1997). Juveniles may therefore need a longer period than adults to accumulate the energy required to molt and grow new hair, leading to a delayed molt as observed in many species of bats (e.g., *Myotis* sp., *Pipistrellus *sp.; Fraser et al., 2013) and in Cape hare (*Lepus capensis;* Lu, 2003). Reproductive females may also delay winter coat shedding because of the high energy expenditures allocated to maternal care (Cowan & Raddi, 1972; Pérez‐Barbería & Nores, 1996) and the secretion of prolactin (Heydon et al., 1995). High testosterone levels can also inhibit molt (Beltran et al., 2018; Zimova et al., 2018). During pregnancy, females’ testosterone levels are expected to peak just before giving birth (Pavitt, Pemberton, Kruuk, & Walling, 2016), which could delay the onset of molt. Furthermore, individual dominance in gregarious species is often linked with access to resources and body condition (Appleby, 1980; Hamel & Côté, 2009), and thereby, we could expect subordinate individuals to complete winter coat shedding later than dominants. Consequently, numerous sources of individual variation within a population can lead to among‐individual variation in the timing of molt (Cowan & Raddi, 1972; Macdonald & Stewart, 1997).

Here, we used data collected over 25 years in a mountain goat population to assess the role of individual traits and environmental conditions on the timing of molt. In this species, sex, age, and reproductive status have been shown to affect winter coat shedding: Adult males shed their coat earlier than adult females, whereas juveniles (i.e., 1‐ and 2‐year‐old) molt later than adults of the same sex, and lactating females complete molt later than barren females (Chadwick, 2002; Côté & Festa‐Bianchet, 2003). Whether the timing of molt varies among adults of different life stages (i.e., prime age vs. senescent), however, is unknown. Furthermore, whether molt timing is influenced by body mass, social rank, and peak in vegetation quality, which are all linked to energy availability, is also unknown. We hypothesized that body condition, determined by intrinsic traits such as sex, age, reproductive status, and body mass, as well as extrinsic traits such as vegetation phenology, determines the timing of winter coat shedding in mountain goats. We predicted molt date to occur later in years when the peak in vegetation quality is delayed. We also expected to find a nonlinear relationship between molt date and age, anticipating a U‐shaped pattern where molt date is late in juveniles and becomes earlier in young adults, and then increases again as individuals become older due to senescence, that is, the gradual deterioration of functions after maturation (Hamilton, 1966; Williams, 1957; see also Gaillard, Festa‐Bianchet, Yoccoz, Loison, & Toïgo, 2000 for other traits showing nonlinear relationships with age in mammals). Furthermore, we predicted that individuals in better condition, that is, with a higher age‐specific body mass, would molt earlier due to their potential to allocate more resources to pelage growth compared with same‐aged individuals in poorer condition. We anticipated that dominant females would molt earlier than subordinates because dominance could increase access to food resources (Appleby, 1980). Finally, we also investigated the effect of temperature on molt date, anticipating that colder temperatures would delay molt. To our knowledge, no study has assessed the influence of multiple drivers to identify the main mechanisms underlying molt processes, particularly in large mammalian herbivores. Based on an exceptional longitudinal data set, we demonstrate that access to both intrinsic and extrinsic resources is the fundamental driver of molt timing in mountain goats.

## 2 METHODS

### 2.1 Study area and population

The Caw Ridge study area occupies ca. 28 km^2^ in the foothills of the Canadian Rockies in west‐central Alberta, Canada (54°N 119°W). Surrounded by boreal forest, the habitat consists of subalpine forest and alpine tundra, with steep slopes and escape terrains composed of a few rocky cliffs. Mountain goat forage is dominated by grasses and alpine forbs in the summer (Hamel & Côté, 2009). Winter is cold and long, whereas summer is short and may be interrupted by snowfalls at any time (Festa‐Bianchet & Côté, 2008).

Starting in 1989, almost all individuals in the Caw Ridge population have been trapped using self‐tripping Clover traps and remotely controlled Stevenson's box traps baited with salt. All individuals were aged, sexed, and identified with collars and ear tag combinations at one year of age, except for a few individuals at the beginning of the study that were aged by counting horn annuli up to 7 years of age (Côté, Festa‐Bianchet, & Smith, 1998; Stevens & Houston, 1989). All goats were weighed with a spring scale (0.5 kg) at handling, but after 1995, most females were only trapped as yearlings and two‐year‐olds to avoid the risk of kid abandonment (Côté, Festa‐Bianchet, & Fournier, 1998). We collected additional masses of individuals (mostly adult females) without handling individuals using remotely controlled electronic platform scales (0.5 kg) baited with salt (Festa‐Bianchet & Côté, 2008). All applicable guidelines of Canada, Alberta, and Université Laval for the care, welfare, and use of animals were followed.

### 2.2 Individual characteristics

Between 1989 and 2016 from mid‐May to mid‐September, we observed individuals using spotting scopes (15–40×; distances ranging between 200 and 700 m) on a daily basis, weather permitting. Mountain goats molt only once a year (Côté & Festa‐Bianchet, 2003; Holroyd, 1967). Their summer coat consists of guard hairs (>5 cm) that start to grow as soon as they start molting. This guard hair layer will continue to grow, often until reaching over 20 cm by November or early December. An insulating underlayer of wool will also grow 3 to 5 cm thick during this period (Côté & Festa‐Bianchet, 2003). Mountain goats usually shed their winter coat symmetrically on both sides, beginning from the head and continuing progressively to the chest, forelegs, sides, back, and back legs, with the rump and the belly being the last areas to molt. To determine the date an individual finished molting, we recorded at each observation the percentage of the winter coat shed by each individual. This was estimated using 5% shedding classes. Molt date was the day when winter coat shedding reached 99%–100%. Molt date was not recorded in 1998, 1999, and 2001.

For adult females, we had sufficient data on reproductive status, social rank, and body mass to assess the influence of these variables on molt timing. In this population, females produce singletons, and we determined the reproductive status of females each year by the observation of the presence/absence of a kid as well as nursing behavior (Festa‐Bianchet & Côté, 2008). To determine annual social rank of adult females, we collected ad libitum agonistic interactions (Altmann, 1974), recording for each dyad the winner and loser, determined by the opponent's withdrawal (Côté, 2000). Each year, we ranked adult females in a dominance hierarchy that was tested for linearity according to de Vries (1998). All yearly dominance hierarchies were linear (all *h*’ values ≥0.2, all *p* values <0.001), and thus, females were ranked each year in the linear hierarchy (de Vries, 1998). We accounted for annual variation in the number of adult females by converting the rank between 0 (subordinate) and 1 (dominant) as follows: standardized rank = 1–rank/number of adult females in a given year (Côté, 2000). Because female age is strongly correlated with social rank (*r* > 0.9; Côté, 2000), we determined age‐specific social rank for each female each year using the residuals from a polynomial regression of social rank on age (Théoret‐Gosselin, Hamel, & Côté, 2015).

Because we recorded several masses throughout the summer, we adjusted female body mass to July 15th using the average daily summer mass gain for five age classes of females (3, 4, 5, 6, and ≥7 years old; see Hamel, Côté, & Festa‐Bianchet, 2010). For each class, we ran a linear mixed model (LMM) using the “lmer” function from the “lme4” package (Bates, 2015) in R (R Core Team, 2016). We fitted individual identity and year as random intercepts and reproductive status as a fixed covariate, and then chose the best polynomial regression to fit the relationship with Julian day (Hamel et al., 2010). Furthermore, because body mass is correlated with age and we wanted to evaluate the influence of body mass independently of age, we calculated age‐specific body masses. Since mass increases with age until approximately 7 years of age (Côté & Festa‐Bianchet, 2001), we calculated age‐specific masses as the residuals of the cubic regression of mass on age using age as a continuous variable from 3 to 15, where age 15 included all females 15 years and older to have a minimum of 5 individuals in the oldest age class. Body mass therefore represents females that are heavier or lighter for a specific age.

### 2.3 Environmental conditions

**Figure A1:**
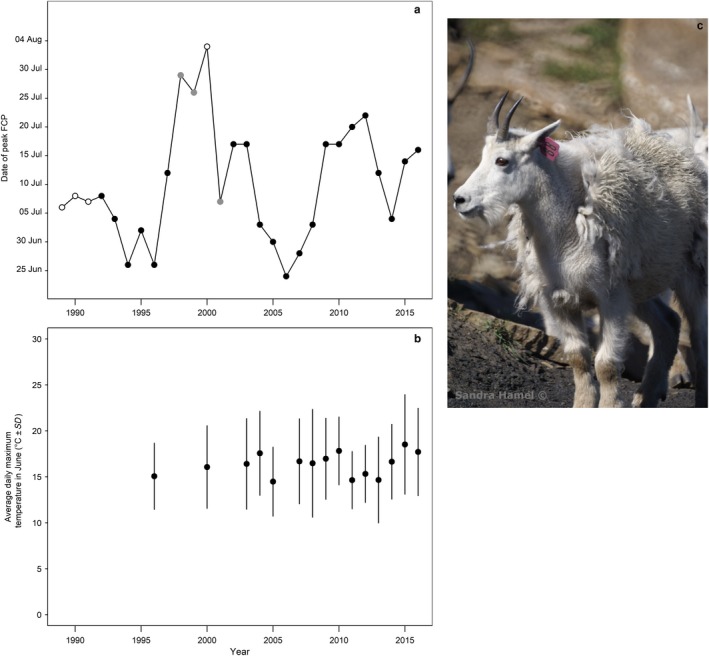
Annual variation (a) in the date of peak fecal crude protein (FCP) at Caw Ridge (AB, Canada, 1989–2016), and (b) in the average daily maximum temperature recorded in June at Hendrickson Creek's weather station close to Caw Ridge (AB, Canada, 1996–2016). A: Filled dots represent date estimated with FCP, whereas open dots represent dates estimated from the relationship between FCP and Integrated Normalized Difference Vegetation Index (see Methods; Hamel, Côté, Côté, Gaillard, & Festa‐Bianchet, 2009; Hamel, Garel et al., 2009). Gray dots represent years excluded from analyses because no molt date was recorded. B: Filled dots are the averages, and bars show the standard deviations (*SD*). (c) A yearling mountain goat male that is partially molted (Caw Ridge, August 2008). Photographed by Sandra Hamel

To determine annual variation in resources availability, we estimated the date at which vegetation quality peaked based on fecal crude protein (hereafter named “FCP”; Hamel, Garel, Garel, Festa‐Bianchet, Gaillard, & Côté, 2009). Each year from mid‐May to mid‐September, we collected 5 to 12 fresh fecal samples every 2 to 3 weeks. We air‐dried samples in paper bags and then assessed the percentage protein content in each sample using the macro‐Kjeldahl acid digestion procedure (AOAC, 1984). For each year, we determined the date of the peak in FCP from the relationship between date and the natural logarithm of FCP using a cubic spline smoother (Blanchard, Festa‐Bianchet, Gaillard, & Jorgenson, 2003; Hamel, Garel et al., 2009). We did not evaluate the peak in FCP in years when fecal samples were not collected (1989 to 1991) or when the peak in FCP could not be reliably estimated because too few samples were collected (in 2000). Nonetheless, we have shown in this population that the peak in FCP correlates strongly (*r* = −0.81) with the Integrated Normalized Difference Vegetation Index in June (INDVI; Hamel & Côté^2009^), and hence, we used the relationship between INDVI and FCP to obtain a predicted date for the peak FCP for these four missing years. The date of the peak in FCP showed wide variation during the 28 years of the study, with late peaks representing delayed springs compared with early peaks (Figure A1a in Appendix; Hamel & Côté^2009^).

To investigate the effect of temperature on molt date, we computed the average daily maximum temperature in June. The onset of molt in mountain goats typically occurs in June, and males sometimes complete shedding by early July. Therefore, it would be unlikely that temperature variable after June would modulate molt timing, especially in males. We used daily maximum temperature data from the Environment Canada weather station at Hendrickson creek, which is the closest station with the least missing values (65 km southeast, 1,448 m a.s.l., 53.80 N, 118.45 W). Daily maximum temperatures in June were correlated with those recorded directly on the study site (*r* [95% CI] = 0.93 [0.91; 0.94], *n* = 713). We used data from the meteorological station because it included much fewer missing values than field data. We computed the average maximum temperature in June only for years with at least 25 daily maximum temperatures recorded, that is, 15 out of 25 years (Figure A1b in Appendix).

### 2.4 Statistical analyses

Forty individuals (3.3%) did not complete molt before the end of the field season. We explored how these individuals were distributed according to sex, age, and reproductive status (Table A1 in Appendix) and found that most individuals molting after the end of the field season were juveniles and adult barren females (Table A2 in Appendix). Excluding these individuals would bias estimates, especially for assessing the influence of reproductive status because barren females are known to molt earlier than lactating females. Therefore, we included these individuals and attributed September 10th (i.e., last molt date recorded) as their molt date to minimize bias. We converted all dates to Julian days starting on June 1st.

To evaluate the influence of individual traits, vegetation quality, and temperature on molt date, we fitted a LMM (package “lme4”; Bates, 2015) using individual identity as a random intercept. To evaluate the influence of the different variables on molt date, we used four successive LMM because some variables were not available for some groups of individuals (see below). The first model included all molt dates (*N* = 1671 on 354 individuals) and included age, sex, and their interaction, as well as date of peak FCP and its interaction with sex (Table 1). We modeled age using a B‐spline (package “splines” in R) and peak date in FCP as a quadratic effect to account for the nonlinear influences of these variables on molt date. We used likelihood ratio tests to determine the best polynomial degree of the spline function and the relevance of the quadratic effect, retaining the simplest model when different models provided equivalent support. In this first LMM, support was for a model with age fitted with a degree of 5 and a quadratic effect of date of peak FCP. In addition, we grouped males 12 years and older and females 15 years and older together to ensure the last age class included a minimum of 5 individuals. To meet the normality assumption, molt dates were power transformed, with the power *λ *= −0.3 according to a Box–Cox evaluation (Box & Cox, 1964). With the variances obtained from this first LMM, we used the function “rpt” (package “rptR”; Stoffel, Nakagawa, & Schielzeth, 2017) to estimate the average repeatability of molt date in mountain goats. As defined by Nakagawa and Schielzeth (2010), repeatability represents the proportion of the total variance due to differences among individuals.

**Table 1:** Influences of environmental and intrinsic variables on the molt date of mountain goats at Caw Ridge (AB, Canada), 1989―2016, with influential variables highlighted in bold

Model	Variable^a^	*df*	*F*
Model 1	Intercept	1	208.1
	**Age**	**5**	**108.6**
	**FCP**	**2**	**70.4**
	**Sex**	**1**	**316.2**
	**Sex × age**	**5**	**85.5**
	Sex × FCP	2	8.6
Variance	Random intercept ID	0.00005	
	Residuals	0.00016	
Model 2	Intercept	1	36.4
	**Age**	**5**	**77.2**
	**FCP**	**2**	**33.1**
	**Sex**	**1**	**278.6**
	**Sex × age**	**5**	**50.9**
	Sex × FCP	2	9.9
	**Temperature**	**1**	**10.1**
Variance	Random intercept ID	0.00005	
	Residuals	0.00018	
Model 3	Intercept	1	70.2
	**Age**	**1**	**131.5**
	**FCP**	**1**	**61.3**
	Social rank	1	2.1
	**Reproductive status**	**1**	**89.5**
	**Reproductive status × age**	**1**	**0.6**
Variance	Random intercept ID	0.00002	
	Residuals	0.00043	
Model 4	Intercept	1	63.2
	**Age**	**1**	**129.3**
	**FCP**	**1**	**42.8**
	Social rank	1	0.3
	**Reproductive status**	**1**	**51.3**
	**Mass**	**1**	**30.4**
	Mass × social rank	1	0.3
	**Mass × reproductive status**	**1**	**6.1**
Variance	Random intercept ID	0.00004	
	Residuals	0.00063	

a *t*‐values are presented for intercepts. FCP: date of peak fecal crude protein. ID: individual identity. Temperature: average daily maximum temperature in June (°C).

We assessed the influence of temperature in a separate model because the average maximum daily temperature in June was only available for 15 out of the 25 years (*N* = 1,100 on 295 individuals). This second LMM was fitted exactly the same as the first one but also included the effect of June average maximum temperature.

**Figure 1:**
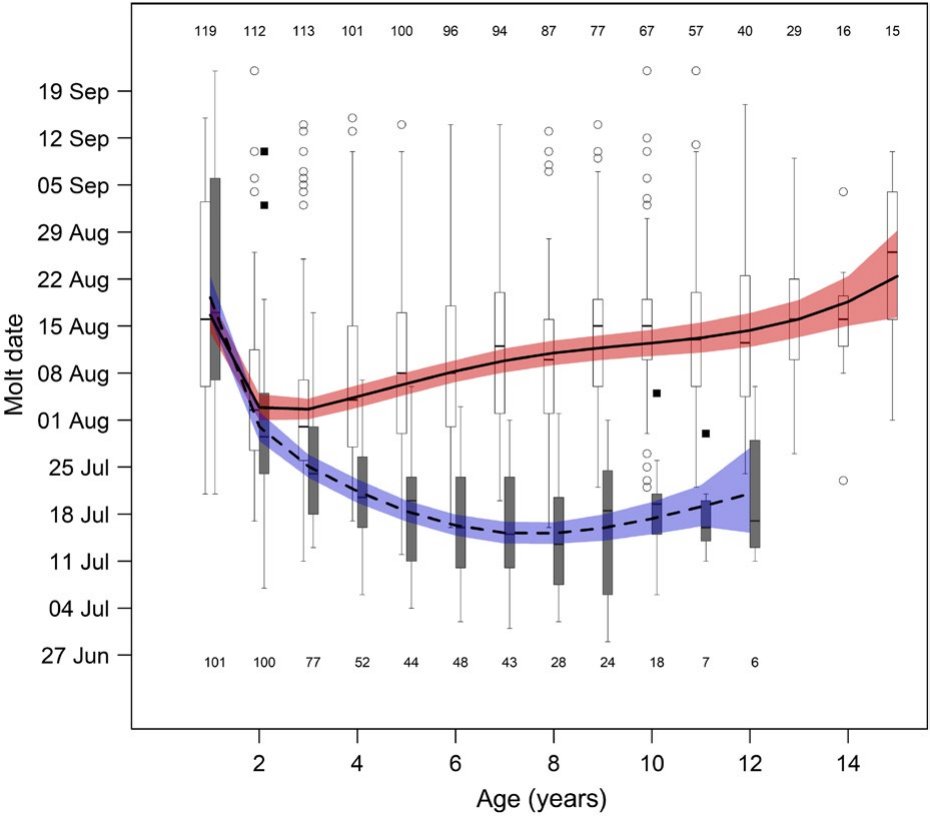
Effects of age and sex on molt date of mountain goats at Caw Ridge (AB, Canada), 1989―2016. Curves represent model predictions and 95% confidence intervals (females: solid curve and red shadow; males: dashed curve and blue shadow), whereas box plots at each age present the raw data (females: white boxes; males: gray boxes). The lines in the boxes represent medians, and boxes represent lower to upper quartile ranges, with whiskers extending up to 1.5 times the interquartile range and data beyond that distance represented by dots (females) and squares (males). Numbers represent sample size at each age for females (top) and males (bottom)

**Figure 2:**
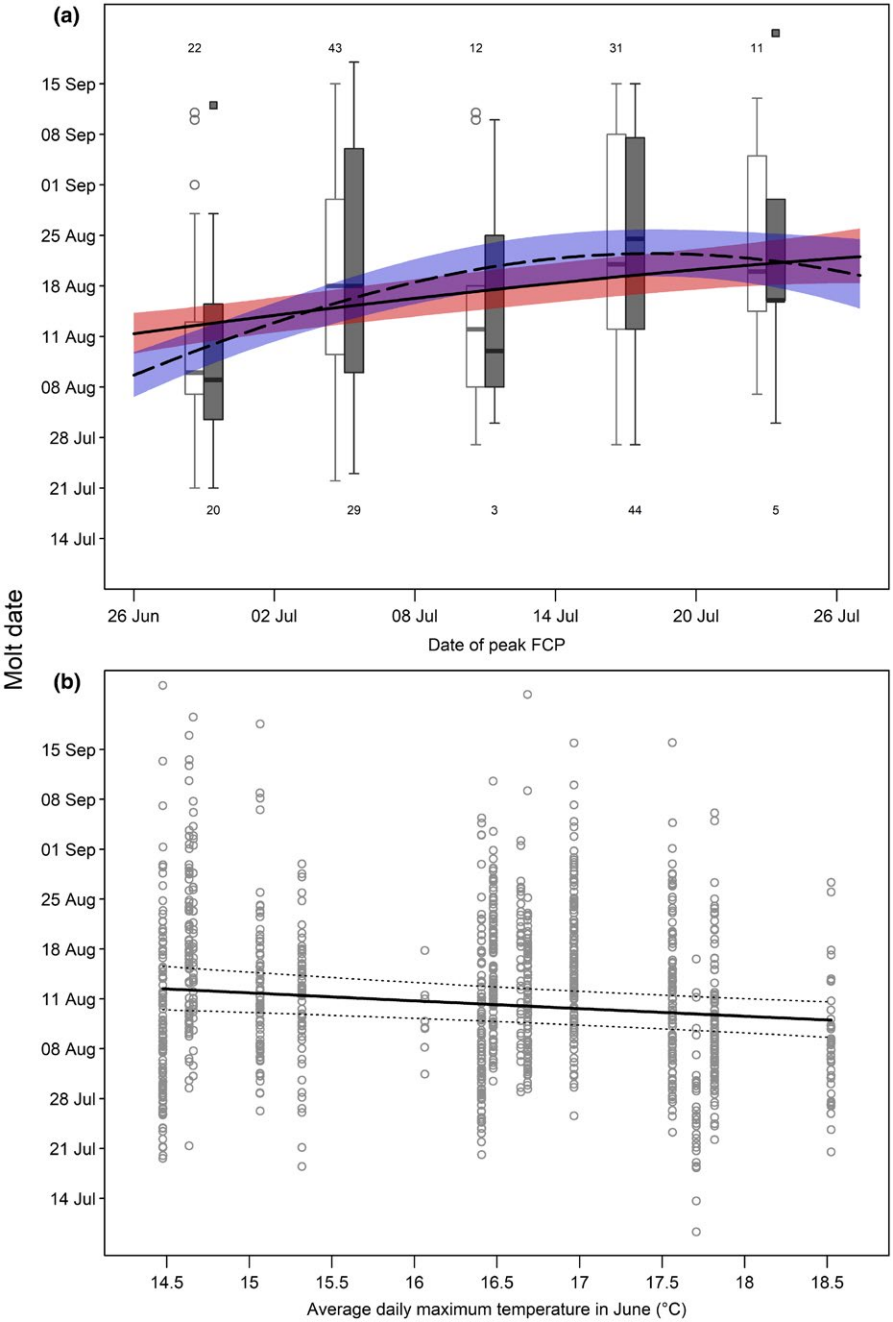
Effect of the timing in peak fecal crude protein (FCP) on (a) and the average daily maximum temperature in June on (b), on molt date of mountain goats at Caw Ridge (AB, Canada), 1989―2016. (a) The curves represent model predictions at age 1 and 95% confidence intervals for females (solid curve and red shadow) and males (dashed curve and blue shadow). The raw data are presented in box plots (see Figure 1 for definition of symbols) for different intervals of peak date in FCP (females: open dots and white boxes; males: solid squares and gray boxes). Numbers represent sample size for each FCP interval in yearling females (top) and yearling males (bottom). (b) The curve represents model predictions for a female at age 5, and dashed lines represent 95% confidence intervals. The dots are the partial residuals controlling for the influence of the other variables included in analysis

Because reproductive status and social rank were only available for adult females, we fitted a third LMM including only molt dates of females ≥3 years of age to evaluate the influence of these two variables (*N* = 834 on 155 individuals). We built a LMM similar to the first one, including the main effects of the first LMM (i.e., age and date of peak FCP) but testing in addition the effects of social rank, reproductive status, and the interaction between reproductive status and age (Table 1). We did not fit nonlinear effects for age and date of peak FCP in this third model because the reduced data set including only adult females showed a linear effect for these parameters (see Figures 1 and 2). In this model, we used *λ* = −0.6 to meet the normality assumption.

Lastly, because not all goats were weighed each year and most masses available were for adult females, we built a fourth LMM including only molt dates of females ≥3 years for which we had measured mass (*N* = 514 on 128 individuals). The fourth LMM was built similarly as the third model, including the main effects of the third LMM as well as body mass and its interaction with both social rank and reproductive status (Table 1). In this LMM, we used a *λ* = −0.5 to meet the normality assumption. All analyses were run using version 3.3.2 of the R statistical software (R Core Team, 2016).

## 3 RESULTS

The first model assessing molt date for the whole population confirmed the influence of age and sex on molt date, with age showing a nonlinear effect that varied between sexes (Table 1; Figure 1). Molt date did not differ between sexes in yearlings and 2‐year‐olds, but adult males molted earlier than females, with up to a one‐month difference at prime age (Figure 1). In both sexes, molt date was later in juveniles, decreased in adults, and then increased again in older individuals, but this U‐shaped pattern differed between sexes (Figure 1). Female molt date occurred earliest at 2 and 3 years old and then showed a consistent increase in molt date with age, whereas molt date in males declined progressively from age 1 until reaching a minimum at prime age between 6 and 9 years old, and then showed an increase at older ages. In addition, this model highlighted the positive influence of environmental conditions (Table 1): Males and females molted up to two weeks earlier when the peak in vegetation quality occurred earlier, with little difference between sexes (Figure 2a). Based on this first model, repeatability of molt date (R [95% CI]) was estimated at 0.25 [0.19; 0.30]), which is within the range of the repeatabilities considered as moderate by Nakagawa and Schielzeth (2010).

**Figure 3:**
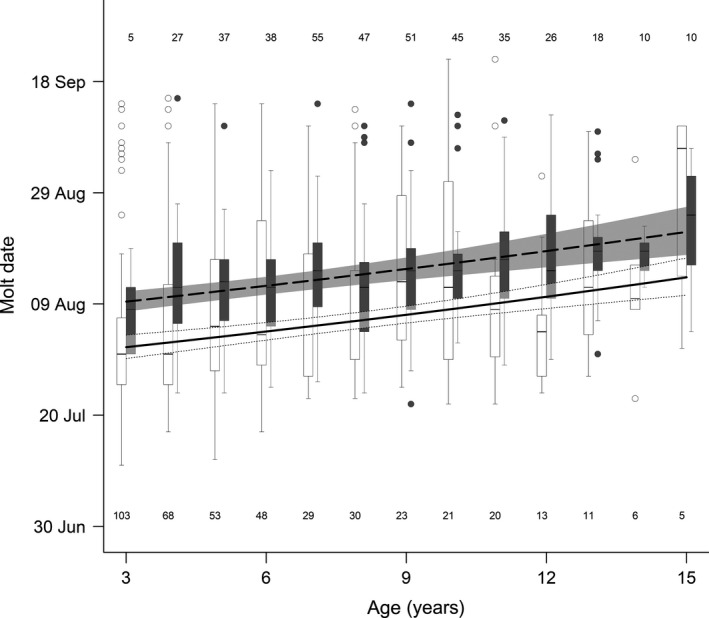
Effect of reproductive status on molt date in adult female mountain goats at Caw Ridge (AB, Canada), 1989―2016. Curves represent model predictions and 95% confidence intervals (barren: solid curve and white shadow; lactating: dashed curve and gray shadow), whereas box plots at each age present the raw data (barren: open dots and white boxes; lactating: solid dots and gray boxes; see Figure 1 for definition of symbols). Numbers represent sample size at each age for barren females (bottom) and lactating females (top)

**Figure 4:**
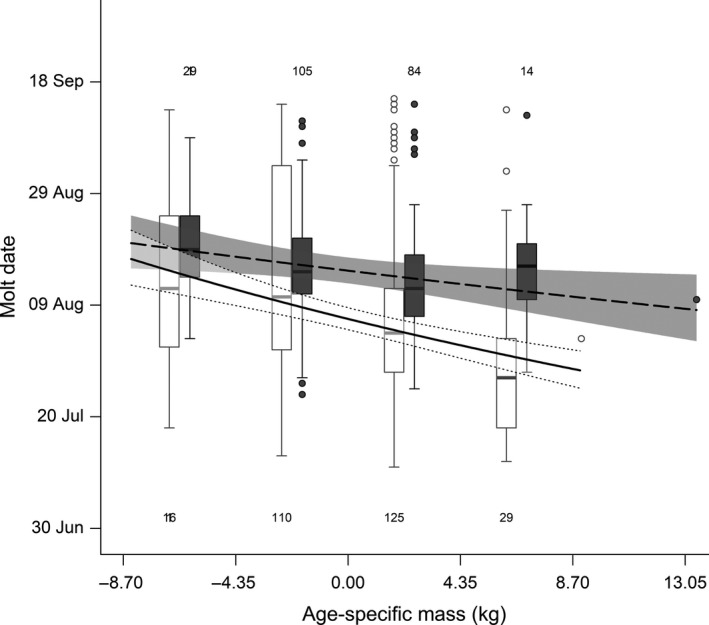
Effect of reproductive status and age‐specific mass on molt date in adult female mountain goats at Caw Ridge (AB, Canada), 1989―2016. Curves represent model predictions and 95% confidence intervals (barren: solid curve and white shadow; lactating: dashed curve and gray shadow). The raw data are presented in box plots (see Figure 1 for definition of symbols) for different intervals of age‐specific mass (barren: open dots and white boxes; lactating: solid dots and gray boxes), with the last mass interval (no box plot shown) including only one value for each reproductive status. Numbers represent sample size for each mass interval in barren females (bottom) and lactating females (top)

The second model including the average daily maximum temperature in June (Table 1) showed that a decrease of 4°C tended to delay molt date by 4 days (Figure 2b). The third model focusing exclusively on adult females showed an influence of reproductive status on molt date (Table 1), with females having an offspring molting about 10 days later than barren females, irrespective of age (Figure 3). Social rank, however, did not influence molt date (Table 1): Coat shedding ended at a similar date for subordinate and dominant females of the same age (estimate [95% CI]: 0.005 [−0.002; 0.012]). The last model that included only adult females for which we had information on body mass indicated an influence of body condition that varied between lactating and barren females (Table 1). Molt date became earlier with improvement in body condition, but more importantly for barren than lactating females (Figure 4). Compared with females in poor condition for which reproductive status did not affect molt date, females in good condition molted about 5 days earlier if they were lactating and 15 days earlier if they were barren (Figure 4). This highlights that the earlier molt of 10 days found in barren females (Figure 3) mostly occurred in females in relatively good condition (Figure 4). The influence of body mass did not vary with females of different social ranks (estimate [95% CI]: 0.0009 [−0.0019; 0.0004]; Table 1).

## 4 DISCUSSION

Our results greatly extended knowledge on the mechanisms explaining the timing of molt in wild herbivores by showing for the first time that coat shedding is tightly linked to resources availability, both in terms of individual body condition and access to food. Interestingly, although it has been shown previously that lactating females molt later than barren females as a result of the additional energy allocated for maternal care (e.g., black‐tailed deer (*Odocoileus hemionus*; Cowan & Raddi, 1972) and Cantabrian chamois (*Rupicapra pyrenaica parva*; Pérez‐Barbería & Nores, 1996)) and hormonal changes (e.g., red deer; *Cervus elaphus; *Heydon et al., 1995), we showed for the first time that this difference only occurs for females in good condition. Females in poor condition had a similar molt date whether or not they produced a kid, suggesting that growth of new hairs in ungulates is first limited by energy reserves, and then by allocation to maternal care. The influence of body condition on molt progression was also reported in male European badgers (*Meles meles*), where high levels of testosterone and lower body condition delayed molt (Macdonald & Stewart, 1997). In our study, molt date could also be driven by hormonal changes, but we do not have hormonal data to assess this. As reviewed by Beltran et al. (2018), high testosterone levels at the end of gestation have been shown to inhibit molt onset in breeding females. In several biannual molting mammals, however, an increase in prolactin secretion during lactation has been shown to either trigger spring molt or inhibit fall molt (Heydon et al., 1995; Zimova et al., 2018). More work is needed to better understand the influence of hormonal drivers on molt timing in herbivores.

In addition to body condition, late access to high‐quality vegetation, which limits direct energy acquisition, delayed molt substantially in both sexes. Some previous studies also suggested that molt completion was timed with food abundance (Danner et al., 2015; Wolfe et al., 2017) and access to high‐quality food resources (Lu, 2003), especially in poor condition individuals (Macdonald & Stewart, 1997). The review by Ling (1970) also reported that malnutrition delayed molt in various wild mammals (e.g., black‐tailed deer, caribou (*Rangifer tarandus groenlandicus*), and elephant seal (*Mirounga leonida*)). Moreover, temperature is another external cue that can interact with photoperiod to modulate the timing of molt (Beltran et al., 2018; Zimova et al., 2018). Colder temperatures can delay molt completion, a tendency we observed for mountain goats. Our findings corroborate earlier research and provide support to the hypothesis that, in highly seasonal environments with short periods of food availability such as temperate and alpine regions, molt processes are modulated by access to resources, vegetation phenology, and temperature.

Apart from females that were old or in poor condition, juveniles of both sexes showed the latest molt dates. The absence of a sex difference in juveniles’ molt date could be related to similar allocation of energy reserves to growth, because sexual dimorphism in body mass develops more importantly from 2 years old onward (Côté & Festa‐Bianchet, 2003; Festa‐Bianchet & Côté, 2008). Moreover, the growth rate of yearlings is much steeper relative to their body size than that for 2‐year‐olds, indicating that they likely need to allocate more energy to growth (Festa‐Bianchet & Côté, 2008; Gaillard et al., 1997). A delay in hair growth and molt timing was also reported in juveniles of various bat species (Fraser et al., 2013), collared lemmings (*Dicrostonyx groenlandicus*; Zimova et al., 2018), hares (Lu, 2003; Zimova et al., 2018), racoons (*Procyon lotor*; Hanni & Millar, 1993), and arctic foxes (*Vulpes lagopus*; Zimova et al., 2018). In addition, growth rate was shown to delay age at molt completion in younger red‐backed voles (*Myodes gapperi*), suggesting a trade‐off between molt and growth in early life stages (Sare, Millar, & Longstaffe, 2005). Our results also agree with the delayed molt previously documented in juvenile mountain goats (Chadwick, 2002; Côté & Festa‐Bianchet, 2003).

With respect to later life stages, our study provides a novel and considerable insight on timing of molt with age. Our exceptional longitudinal data set allowed us to show that changes in molt date with age are nonlinear, with considerable delays at older ages, and that molt timing contrast importantly between sexes after the juvenile stage. Sexual differences in molt have been reported in birds such as Cassin's auklet (*Ptychoramphus aleuticus*) and two species of ptarmigans, with males starting to molt earlier than females (Emslie, Henderson, & Ainley, 1990; Zimova et al., 2018). In mammals, the effect of sex differs among species (Zimova et al., 2018). For example, Hanni and Millar (1993) found no effect of sex in racoons, although male mountain hares, arctic hares, collared lemmings, and siberian hamsters molt earlier in fall (Zimova et al., 2018). We showed that prime‐age males molt up to one month earlier than prime‐age females, a difference that could be explained by differential habitat selection and foraging strategies between sexes. Male mountain goats are often observed near or below the treeline compared with females, whose strategy is to use higher elevations and to stay close to escape terrain where predation risk is lower (Côté & Festa‐Bianchet, 2003; Hamel & Côté, 2007). Thus, males likely surf on the altitudinal gradient of vegetation quality (Merkle et al., 2016), allowing them to forage on a wider elevation range that provides access to better forage quality earlier and for a longer period than nursery groups.

Additionally, differences in energetic constraints between the sexes and temporal variation of gonadal hormones could explain the sex difference in molt date observed. First, females usually face higher energetic constraints than males because they must recover from the energetic costs of lactation. Secondly, gonadal hormones do not peak at the same times for males and females. In ungulates especially, female testosterone levels peak typically in spring, before giving birth (Pavitt et al., 2016; Shargal et al., 2008), whereas male testosterone levels peak before or during rut (Pelletier, Bauman, & Festa‐Bianchet, 2003; Schams & Barth, 1982). This temporal variation of gonadal hormone levels likely explains why males either molt later in spring or earlier in fall in several biannual molting mammals (Zimova et al., 2018). Consequently, although adult male mountain goats likely acquire faster the energy needed for pelage growth in the summer that could allow them to molt much earlier than adult females, sex differences in hormonal levels and energy constraints may also influence molt completion in this species.

What is even more notable is the difference in the pattern of molt timing with age between sexes. For female mountain goats, earliest molt dates occurred at ages 2 and 3, and molt dates started to occur progressively later at age 4, which is the minimum age at first reproduction (except for 0.4% of females that reproduced at age 3). Therefore, the delay in molt date with age observed in females supports the prediction that reproductive maturation should mark the onset of senescence (Hamilton, 1966; Williams, 1957). Males, however, progressively reached earlier molt dates from the juvenile stage until ages 7 or 8, which is the time of peak reproduction (Mainguy & Côté, 2008). After the peak in reproduction, male molt dates started to be delayed again. Delayed molt in males might reflect senescence occurring after 8 years old because this is when the first decline in pre‐rut body mass is observed, likely as a result of intense allocation to reproduction at that age (Mainguy & Côté, 2008). With increases in reproduction probability, and concomitantly allocation to reproduction, males experience higher energetic costs from changes in their activity budget during the rut, thereby reducing their body condition in fall and winter (Forsyth, Duncan, Tustin, & Gaillard, 2005; Mainguy & Côté, 2008; Pelletier, Mainguy, & Côté, 2009). Old males might therefore need to recover from energetic costs of the rut, hence needing more time to accumulate sufficient energy reserves to grow new hairs and molt their old pelage the following summer. Future studies should aim at collecting sufficient mass data on male ungulates to be able to evaluate this hypothesis.

Overall, our detailed longitudinal data allowed demonstrating for the first time that senescence also occurs for the molting process. As individuals are aging and allocate more energy to reproduction and body maintenance, the accumulation of energy reserves needed to grow hair likely takes longer, thus delaying molt in senescent individuals. This novel finding is considerable because few studies have assessed age‐related changes in physiological traits (Nussey, Froy, Lemaitre, Gaillard, & Austad, 2013), and understanding these changes should help unravel the mechanisms triggering the decline in reproduction and survival performance with age (Monaghan, Charmantier, Nussey, & Ricklefs, 2008). Furthermore, we showed that even though molt timing is moderately repeatable within an individual over the years, individual heterogeneity in energetic reserves modulates molt timing, along with annual variation associated with timing in availability of high‐quality food resources and, to a lesser extent, temperature. Our results therefore suggest that, in herbivores living in temperate and alpine regions, pelage replacement is constrained by environmental phenology affecting the entire population, along with other intrinsic parameters such as body mass limiting molt timing at the individual scale.

## CONFLICT OF INTEREST

The authors declare that they have no conflict of interest.

## AUTHOR CONTRIBUTION

FD, SH, and SDC conceived the design and collected the data. FD and SH performed the analyses and drafted the article. SH and SDC provided critical revision. SDC funded the research. All authors contributed in criticizing, improving the manuscript, designing methodology as much as ideas and questions to test, and provided final approval of the version to be published.

## Data Availability

Data available from the Dryad Digital Repository at https://doi.org/10.5061/dryad.5t165h5 (Déry, Hamel, & Côté, 2019).

## APPENDIX 1

**Table A1:** Log‐linear model of the likelihood of not recording the final molt date in relation with age class (juveniles vs. adults), sex, and their interaction, as well as with reproductive status (lactating vs. barren), in mountain goats at Caw Ridge (AB, Canada), 1989―2016

Model	Variables	*z‐value*	*p‐value*
All individuals (*N* = 1687)	Intercept	−10.5	<0.001
	Age class	−3.5	<0.001
	Sex	−1.9	0.06
	Sex × Age class	0.0	1.000
Adult females (*N* = 896)	Intercept	−13.4	<0.001
	Reproductive status	−2.2	0.03

**Table A2:** Percent recorded and not recorded molt dates by age classes (juveniles: 1‐ and 2‐year‐olds; adults: 3‐year‐olds and over) and sex (F: females; M: males) and by reproductive status (adult females only) in monitored molts of mountain goats at Caw Ridge (AB, Canada), 1989―2016

Age	Sex	Recorded (%)	Not recorded (%)
1–2	F	92.5	8.5
1–2	M	96.0	4.0
3+	F	96.9	3.1
3+	M	100.0	0.0

Reproductive status	Recorded (%)	Not recorded (%)
Lactating	98.2	1.8
Barren	95.6	4.4
